# Whole‐genome resequencing reveals signature of local adaptation and divergence in wild soybean

**DOI:** 10.1111/eva.13480

**Published:** 2022-09-20

**Authors:** Jiao Wang, Zhenbin Hu, Xiliang Liao, Zhiyu Wang, Wei Li, Peipei Zhang, Hao Cheng, Qing Wang, Javaid Akhter Bhat, Hui Wang, Biao Liu, Hengyou Zhang, Fang Huang, Deyue Yu

**Affiliations:** ^1^ National Center for Soybean Improvement, National Key Laboratory of Crop Genetics and Germplasm Enhancement, Jiangsu Collaborative Innovation Center for Modern Crop Production Nanjing Agricultural University Nanjing China; ^2^ Department of Biology Saint Louis University St. Louis Missouri USA; ^3^ Crop Tillage and Cultivation Institute, Heilongjiang Academy of Agricultural Science Harbin China; ^4^ Nanjing Institute of Environmental Sciences Ministry of Ecology and Environment Nanjing China; ^5^ Key Laboratory of Soybean Molecular Design Breeding Northeast Institute of Geography and Agroecology, Chinese Academy of Sciences Harbin China

**Keywords:** genome‐environment association, local adaptation, population divergence, wild soybean

## Abstract

Global climate change has threatened world crop production and food security. Decoding the adaptive genetic basis of wild relatives provides an invaluable genomic resource for climate‐smart crop breedinG. Here, we performed whole‐genome sequencing of 185 diverse wild soybean (*Glycine soja*) accessions collected from three major agro‐ecological zones in China to parse the genomic basis of local adaptation in wild soybean. The population genomic diversity pattern exhibited clear agro‐ecological zone‐based population structure, and multiple environmental factors were observed to contribute to the genetic divergence. Demographic analysis shows that wild soybeans from the three ecological zones diverged about 1 × 10^5^ years ago, and then the effective population sizes have undergone different degrees of expansions. Genome‐environment association identified multiple genes involved in the local adaptation, such as flowering time and temperature‐related genes. A locus containing two adjacent MADS‐box transcription factors on chromosome 19 was identified for multiple environmental factors, and it experienced positive selection that enables the adaptation to high‐latitude environment. This study provides insights into the genetic mechanism of ecological adaptation in wild soybean that may facilitate climate‐resilient soybean breeding.

## 1 INTRODUCTION

Climate change exacerbates the stress in plants due to the sessile nature, which constrains worldwide agricultural production and threatens global food security (Kukal & Irmak, 2018; Zhao et al., 2017). It is an efficient strategy to integrate the adaptive genes into crop cultivars to enhance the productivity of crops. Compared with the cultivated plants, their wild relatives harbour higher genetic diversity and show stronger environmental adaptability (Burgarella et al., 2019). However, the genetic basis of adaptability, as well as how the environments have shaped genomic diversity, has been less understood. Mining the genes associated with environmental adaptation from the wild relatives of crops is critical for understanding the adaptation mechanism and climate‐smart cultivar development for feeding the growing global population (Raza et al., 2019).

Wild soybean (*Glycine soja* Sieb. & Zucc.), the ancestor of cultivated soybean (*Glycine max* [L.] Merr.), is mainly distributed in East Asia, including China, Japan, Korean Peninsula and East Russia (24–53°N, 97–143°E). About 90% of world wild soybean germplasms have been preserved in China (Wang et al., 2001). In China, *G. soja* is mainly distributed in three major ecological zones, including the Southern region (SR), Huanghuai region (HR) and Northeast region (NER), which are corresponding to the major soybean cultivation regions (Gai et al., 2000). *Glycine soja* adapted to various environments from the southern to northeast regions in China, with a wide range of environmental factors such as photoperiod, temperature and precipitation. As a short‐day plant, *G. soja* needs to precisely regulate the flowering time to adapt to the local photoperiod (Xu et al., 2013). Temperature is another factor influencing the distribution of *G. soja* (Wang et al., 2001). Adaptation to high‐latitude or high‐altitude environments requires cold‐tolerance in *G. soja*. Wild soybean grows well under harsh or marginal environments and widely adapts to various environments from low to high latitudes (He et al., 2016; Li et al., 2014). Adapting to the wide range of environments indicates that *G. soja* is rich in adaptive genes, which has been emphasized in various studies exploring the genetic basis of adaptation to some certain environmental factors (Ning et al., 2017; Qi et al., 2014). Identifying the adaptive genes in *G. soja* would provide critical insights into the genetic mechanism and offer useful genomic resources for climate‐smart soybeans breeding (Li et al., 2014; Savolainen et al., 2013).

*Glycine soja* accessions have been sequenced and analysed in various studies, which mainly focuses on the generation of pan‐genome sequences, the identification of genomic regions related to domestication and the evolutionary history of *G. max* (Kim et al., 2021; Li et al., 2014; Valliyodan et al., 2019; Xie et al., 2019; Zhou et al., 2015). However, the population dynamics in response to historical climate change, the impact of geographical and environmental factors on genetic differentiation, and the genetic basis of local adaptation in *G. soja* remains largely unexplored. Several previous studies showed that *G. soja* was limited to southern and central China during the last glacial maximum (LGM) and expanded into the Northeast region after LGM, and that environmental factors contributed more to population differentiation than geographic factors in *G. soja* (He et al., 2016; Leamy et al., 2016). Genomic loci associated with the adaptation to environmental factors in *G. soja* were identified with the SoySNP50K platform at a resolution of 32 SNPs/Mb (million base pair) (Anderson et al., 2016). However, these studies are based on the limited number of genetic markers. Genome‐wide resequencing of wild soybean offers an unprecedented opportunity to understand the evolutionary history and adaptation mechanism of *G. soja*.

In this study, we collected and whole‐genome resequenced 185 *G. soja* accessions from three major ecological zones with contrasting environmental conditions in China. We revealed that the effective population sizes of *G. soja* populations have undergone different degrees of expansions by the population demographic analysis. Genome‐environment association analysis identified the genomic regions and candidate genes related to local adaptation. This study provides insights into the demographic history of *G. soja* and high‐confidence adaptive loci for the improvement of the adaptability of soybean modern cultivars.

## 2 MATERIALS AND METHODS

### 2.1 Plant Materials

**FIGURE 1:**
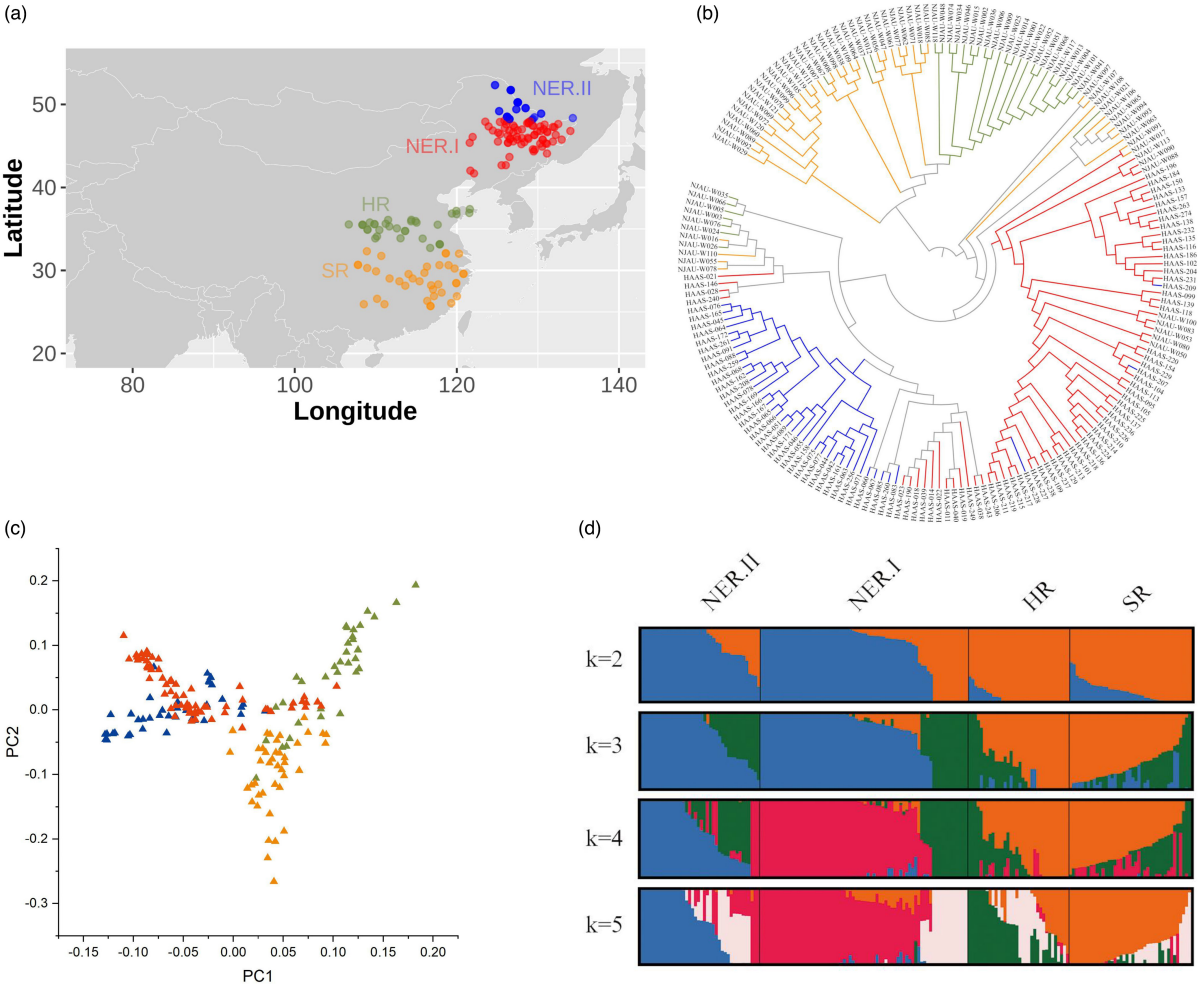
Geographic distribution and population structure of the 185 wild soybean accessions. (a) The geographic distribution of the 185 wild soybean accessions collected from three major ecological zones in China. The blue, red, green and orange dots represent four groups divided according to population structure, viz. NER.II (north of the latitude 48°N of the Northeast region), NER.I (south of latitude 48°N of the Northeast region), HR (Huanghuai region) and SR (Southern region), respectively. (b) Phylogenetic tree of the 185 accessions. Branch colours indicated accessions from different geographical regions, the label indicated the germplasm ID. (c) Principal components analysis of the *Glycine soja*. The colours mean the different groups corresponding to their origins in (a). (d) Model‐based population structure with K from 2 to 5. K means the subpopulation number. The colours indicate the origins of the 185 accessions corresponding to the four different groups.

A total of 185 *Glycine soja* (*G. soja*) accessions were collected from three major ecological zones in China, which correspond to the three major soybean production regions including the Northeast region (NER, 110 accessions), Huanghuai region (HR, 34 accessions) and Southern region (SR, 41 accessions) (Figure 1a, Table S1). The three ecological zones possess diverse environmental conditions, including day length, temperature and precipitation (Table S2) (Xu et al., 1989). For example, the light duration on the summer solstice in the Northeast region (NER), Huanghuai region (HR) and Southern region (SR) is >15 h, 14.5–15 h and < 14.5 h, respectively. Of the panel, 102 accessions originating from Heilongjiang province at NER were provided by the Heilongjiang Academy of Agricultural Sciences (Harbin, China). The remaining 83 accessions from the other provinces were provided by the Germplasm Bank of National Center for Soybean Improvement at Nanjing Agricultural University (Nanjing, China).

### 2.2 DNA sample preparation and sequencing

Genomic DNA was isolated from the fresh leaves of the 185 accessions using the CTAB method (Murray & Thompson, 1980). At least 2 μg genomic DNA from each genotype was used for the sequencing library construction. Paired‐end libraries with insert sizes of 350–400 bp were prepared following Illumina's standard genomic DNA library preparation procedure. Sequencing was performed using the Illumina Hiseq X at the Biozeron Company with 150 bp paired‐end reads (Shanghai, China).

### 2.3 Variants calling and annotation

Raw sequence reads were processed to remove the adaptor contaminants and the low‐quality reads using the Trimmomatic (Version: 0.35) (Bolger et al., 2014). The trimmed reads for the 185 accessions were deposited into the National Center for Biotechnology Information (NCBI) BioProject database with accession number PRJNA597660. The cleaned reads were aligned to the soybean reference genome Williams 82 (assembly v2.0) using Burrows‐Wheeler Aligner (BWA, Version: 0.7.17‐r1188) (Li & Durbin, 2009; Schmutz et al., 2010). The bam files were sorted and indexed using SAMtools (Version: 1.9) (Li & Durbin, 2009). Duplicated reads were marked using the MarkDuplicates function in the Picard package (Version:1.87) (https://github.com/broadinstitute/picard). The sequencing depth and coverage compared with the reference genome were calculated based on the alignments. Single‐nucleotide polymorphisms (SNPs) and short insertion and deletions (Indels) were called using the UnifiedGenotyper function from the Genomic Analysis Toolkit (GATK, Version: 3.3) with default parameters (http://www.broadinstitute.org/gatk/) (DePristo et al., 2011). The SNPs with a high missing rate (>0.5) and low minor allele frequency (MAF) (<0.05) were removed. SNPs and Indels were annotated using ANNOVAR (Version: 2013‐08‐23) (Wang et al., 2010) based on the annotation of the Williams 82 reference genome (Wm82.a2.v1, internal identifier v275 accessed from the Phytozome database).

### 2.4 Alignment on *G. soja* genome

Considering the genomic difference between *G. soja* and *G. max*, the trimmed reads were also mapped to *G. soja* PI 483463 (assembly v1.0 accessed from Soybase database) (Valliyodan et al., 2019) following the same pipeline as described above.

### 2.5 Population genetics analysis and nucleotide diversity

The neighbour‐joining tree was constructed using the PHYLIP software (Version: 3.696) (Feisenstein, 1989). The website iTOL (Interaction Tree Of Life, https://itol.embl.de, Version: 6) was used to visualize the neighbour‐joining tree (Letunic & Bork, 2021). Principal component analysis (PCA) was conducted using the *eigen* function in *R* 4.1.3. Model‐based population structure was performed using the fastStructure program (Version: 1.0) with default setting (Raj et al., 2014), and the results were passed to the *distruct* (Version: 1.1) for visualization (Rosenberg, 2004).

Linkage disequilibrium (LD) decay was evaluated using *PopLDdecay* (Version: 3.40) (Zhang, Dong, et al., 2019; Zhang, Xing, & Lin, 2019). Nucleotide diversity (π) and Tajima's *D* were calculated with a 200‐kb sliding window and 100‐kb step size using VCFtools (Version: 0.1.16) (Danecek et al., 2011).

### 2.6 Isolation‐by‐distance

Geographic distance between accessions was calculated using the *distm* function from the *geosphere* package (Version: 1.5–14) in *R* 4.1.3. Identity‐by‐state (IBS) between accessions was calculated using the *snpgdsIBS* function in the *SNPRelate* package (Zheng et al., 2012) with ld.threshold = 0.8, maf = 0.05, missing.rate = 0.2. The genetic distance was calculated as 1‐IBS. Isolation by distance between geographic distance and log10‐transformed genetic distance was calculated using the mantel test with *mantel.randtest* function in the *ade4* package (Version: 1.7–19) with 9999 permutations.

### 2.7 Demographic history inference

Pairwise sequentially Markovian coalescent (PSMC) model (Version: 0.6.5‐r67) was used to refer to the history of effective population size changes (Li & Durbin, 2011) with selected accessions from each group. Considering the selfing crop, we combined four randomly selected accessions within each group to create pseudo diploid heterozygous genomes by merging the corresponding bam files. The bam file was converted to fastq format using SAMtools (Version: 1.9) and BCFtools (Version: 1.10.2) (Danecek et al., 2021). The fq2psmcfa function from the PSMC package was used to convert the fastq format to the Fasta format. History population size was referred to using the PSMC function from the PSMC package with ‐p ‘4 + 25 × 2 + 4 + 6’, the mutant rate of 1.5 × 10^−8^, and the one generation per year. R custom code was used to visualize the result.

TreeMix (Version: 1.01) was used to construct a maximum likelihood tree for the four groups with two migration events (Pickrell & Pritchard, 2012).

### 2.8 Genome‐environment association analysis

The climatic data were downloaded from Worldclim (https://www.worldclim.org) using the raster package (Version: 3.5–21). Pairwise Spearman correlation coefficients between the 19 factors were calculated. Once the correlation between two variables was larger than 0.8, one of them was removed (Table S3). Finally, five bioclimatic factors (Bio1: annual mean temperature, Bio3: isothermality, Bio12: annual precipitation, Bio13: precipitation of wettest month, Bio15: precipitation seasonality) and three geographic coordinates (latitude, longitude and altitude) were used as a phenotype in genome‐environment association (GEA). GEA was performed using two methods: redundancy analysis (RDA) and latent factor mixed models (LFMM). LFMM was conducted in the LEA package (Version: 3.8.0) (Frichot et al., 2013). The associated SNPs were determined as the top 0.1% significant SNP. The Manhattan plot was drawn using an R package ‘qqman’ (Turner, 2018).

The RDA was performed to demonstrate the associations between genetic variation and environmental factors. We estimated the proportion of genetic variance that is explained by five bioclimatic factors and three geographical factors. The RDA was performed by the *rda* function in the VEGAN package (Version: 2.5). The standard deviation of 3 was used as a cut‐off to identify the outlier adaptive SNPs.

### 2.9 GO term enrichment and extended haplotype homozygosity decay analysis

The gene ontology (GO) enrichment was performed using the *topGO* package (Version: 2.46.0) with the ‘weight01’ algorithm and ‘fisher’ statistic, the significant GO terms were determined with *p* < 0.05. The GO annotation for soybean (Williams 82) genes was downloaded from the Phytozome database (Young et al., 2010).

Extended haplotype homozygosity (EHH) decay was analysed using the REHH package (Version: 2.0.2) (Gautier et al., 2017).

## 3 RESULTS

### 3.1 Genome sequencing and variants annotation

The 185 *G. soja* accessions collected from the three major ecological zones in China were resequenced in purpose to understand the geographic diversity pattern and the genetic basis of local adaptation of wild soybean (Figure 1a, Table S1). A total of 14 billion paired‐end reads were generated with an average sequencing depth of 12× for each accession. The average mapping rate using the Williams 82 as reference genome was 88.88% (Table S4). Considering the genome divergence between *G. soja* and *G. max*, the reads were also mapped to the genome of *G. soja* PI 483463 (Table S4). The average mapping rate was 88.40% which is comparable with that on Williams 82. Considering that the genome of Williams 82 is better annotated and there are more genomic resources than PI 483463, it was used as the reference genome in the following analyses. A total of 5,123,867 high‐quality single‐nucleotide polymorphisms (SNPs), and 3,335,012 insertions and deletions (Indels) were identified and used for further analyses.

The annotations of the SNPs and Indels showed that 80.17% and 66.74% of the SNPs and Indels, respectively, were localized in the intergenic region. By contrast, a small portion of the variation (4.88% of the SNPs and 1.54% of total Indels) were localized in exons (Table S5). There were 138,185 (2.70%) nonsynonymous SNPs and 107,690 (2.10%) synonymous SNPs, which resulted in a nonsynonymous to synonymous substitution ratio of 1.3. This ratio was similar to the previous studies of 1.36 and 1.47 in soybean (Lam et al., 2010; Valliyodan et al., 2016) but was higher than those in other self‐pollinated crops such as sorghum (the value is 1) (Mace et al., 2013). The nonsynonymous SNPs were identified in 35,833 genes. In addition, those SNPs affecting stop codons were also identified, such as 3659 resulting in stop‐codon‐gain in 2755 genes and 413 for stop‐codon‐loss SNPs in 362 genes.

### 3.2 Clear geographic population structure in *G. soja* collections

We asked whether the different environments of three ecological zones cause the divergence of *G. soja*. To examine it, we built a phylogenetic tree for the 185 *G. soja* accessions using the genome‐wide SNPs. The phylogenetic analysis showed that the population was split into two major clades (Figure 1b). One major clade contained the accessions from HR (Huanghuai region) and SR (Southern region), which was further split into two separate groups. Another major clade mainly contained the accessions from NER (Northeast region), with a few mixed accessions from HR and SR, and this indicates the germplasm exchange across the different regions in China. Notably, accessions from the northern Heilongjiang (north of latitude 48°N) are further grouped into a cluster that is separate from the other accessions from southern NER. Therefore, the NER accessions were classified into two groups: one group including 70 accessions from south of latitude 48° N (hereafter NER.I), and another group including 40 accessions from the north of latitude 48° N (hereafter NER.II). These results showed that the *G. soja* accessions were clustered mainly according to their geographical origins (NER.I, NER.II, HR and SR), and suggested that natural selection or local environments is the major driver of the diversity pattern in *G. soja*. Consistently, the principal component analysis (PCA) and model‐based population structure analysis also supported the phylogenetic result (Figure 1c, d).

**FIGURE 2:**
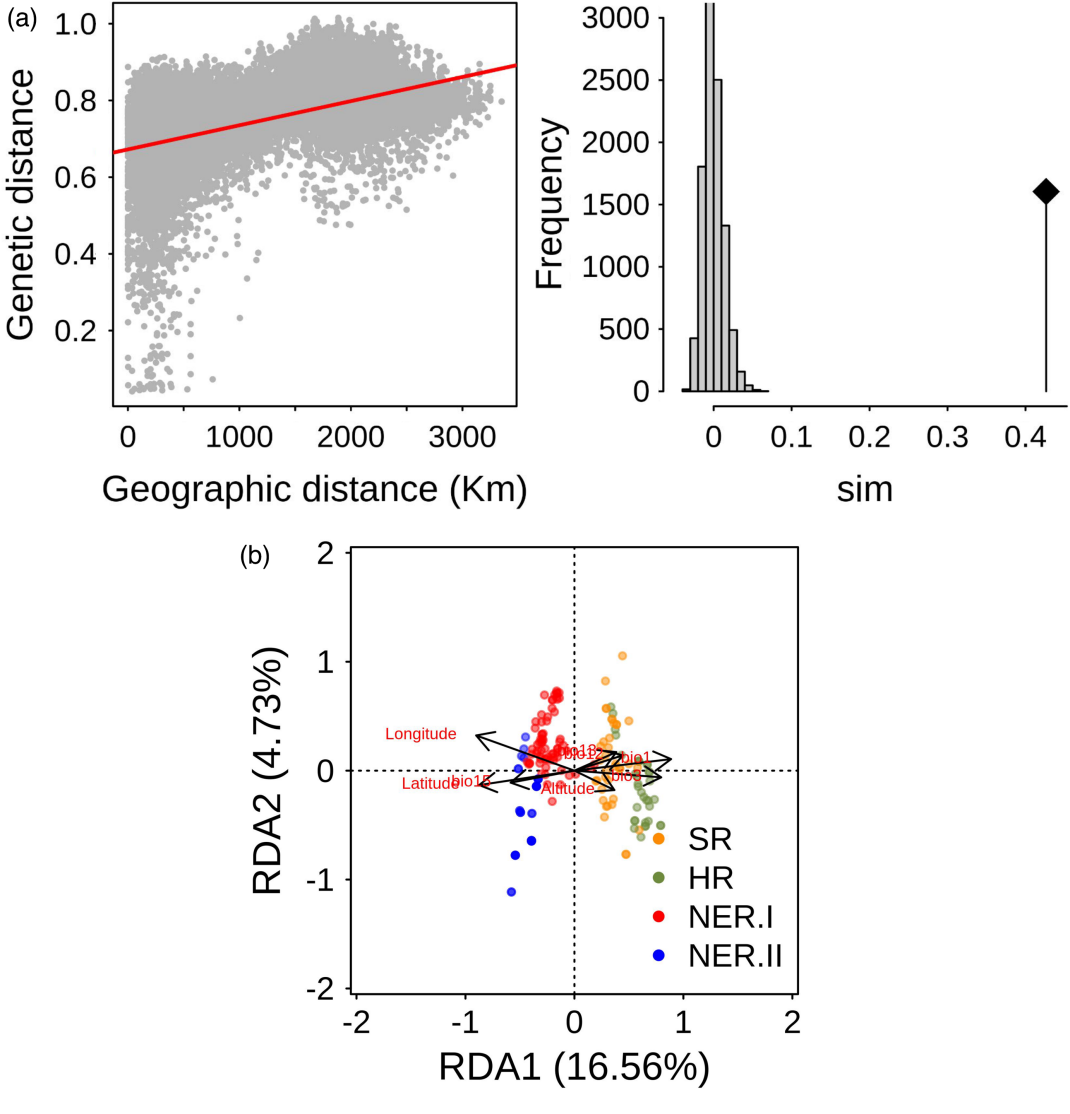
The drivers of the population divergence in *Glycine soja*. (a) The isolation‐by‐distance of the 185 *G. soja* accessions and the significant test using 9999 permutations. (b) Redundancy analysis of the 185 *G. soja* accessions based on five bioclimatic factors (Bio1: annual mean temperature, Bio3: isothermality, Bio12: annual precipitation, Bio13: precipitation of wettest month, Bio15: precipitation seasonality) and three geographic coordinates (latitude, longitude and altitude).

To further analyse the impact of geographic distance on genetic diversity patterns, we calculated the isolation‐by‐distance (IBD) using the mantel test with 9999 times permutation to test the significance. The result showed a significant positive correlation (*r* = 0.51, *p* = 10^−4^) between geographic distance and genetic distance (Figure 2a), suggesting the impact of IBD on genetic diversity patterns in *G. soja*.

To determine the roles of environmental factors on the divergence of *G. soja* population, we performed the redundancy analysis (RDA) using five environmental factors (Bio1: annual mean temperature, Bio3: isothermality, Bio12: annual precipitation, Bio13: precipitation of wettest month, Bio15: precipitation seasonality) and three geographic factors (latitude, longitude and altitude) (Figure 2b). The RDA revealed that 23.98% of the genomic variance could be explained by the environmental factors. The first RDA (RDA1) explained 16.56% of genomic variation in *G. soja*, and it separated the *G. soja* based on their geographic origin; the second RDA (RAD2) explained 4.73% of genomic variations, and it represented the variations within each group. We observed that environmental factors, such as temperature and latitude, played critical roles in the divergence of *G. soja* (Figure 2b). These results indicated that various environmental factors played a critical role in shaping the *G. soja* diversity.

### 3.3 Nucleotide diversity of *G. soja*

We further analysed and compared the genetic diversity (π) amongst the groups to assess whether the geography‐based groups of *G. soja* have different genetic diversity. Overall, we did not observe big differences amongst the groups except for group NER.II, which had the lowest level of nucleotide diversity (π = 1.22 × 10^−3^). Specifically, the HR group had the highest nucleotide diversity (π = 1.40 × 10^−3^), followed by the SR group (π = 1.37 × 10^−3^) and the NER.I group (π = 1.36 × 10^−3^) (Figure S1). The highest genetic diversity in the HR group suggested that the genetic diversity centre of *G. soja* was likely in HR. In addition, the π levels varied greatly in some genomic regions across the genomes and amongst different groups. For example, *G. soja* in HR has higher nucleotide diversity around the 25 Mb (million base pair) region on chromosome 8 (Figure S1). The divergence of the diversity from the geography‐inferred groups implies that natural selection affects the diversity in some genomic regions and these regions might contain variations that are responsible for the local adaptation. Tajima's *D* ranged from 0.86 (NER.II) to 1.21 (NER.I) amongst the four groups. It varied across genomes and diverged at some genomic regions amongst four groups. For example, NER.II group has much lower Tajima's *D* in the region from 18 Mb to 34 Mb on chromosome 4, which suggests NER.II experienced positive selection at this genomic region to adapt to the local environment (Figure S2).

The LD decay reflects natural selection on the genome of a population. To compare the LD decay amongst the different groups, the LD decay was calculated for each group (Figure S3). In the whole population, the LD decay distance with decay to half of its maximum value was 10 kb, which was faster than the results from the previous study (~27 kb) (Zhou et al., 2015). The LD decay speed varied amongst different groups, with the fastest LD attenuation in the SR group, the slowest LD attenuation in the NER.II group. This result was consistent with the diversity pattern where NER.II has the lowest level of genetic diversity whilst SR has a higher level of genetic diversity. These results implied different groups experience different selection pressures.

### 3.4 The demographic history of *G. soja*

**FIGURE 3:**
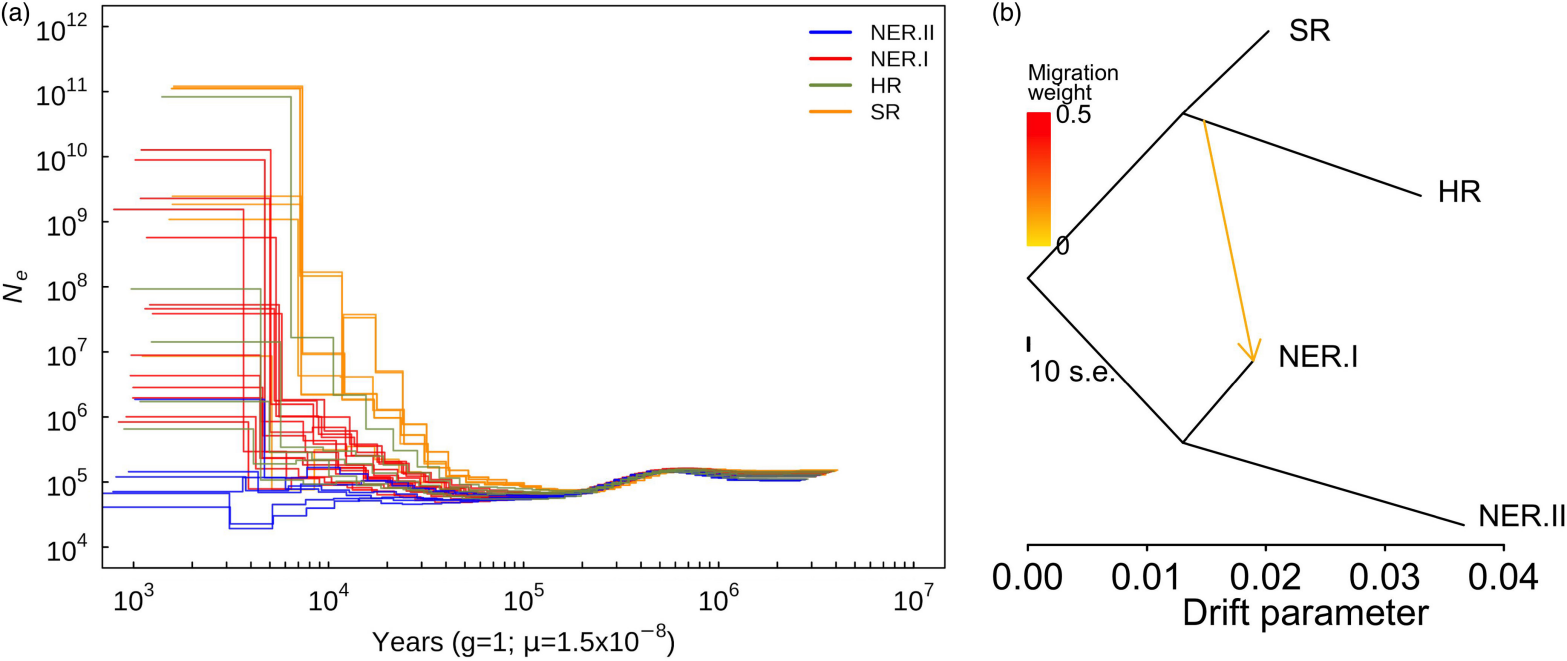
The demographic history of *Glycine soja*. (a) The effective population size of each *G. soja* group inferred by the pairwise sequentially Markovian coalescent (PSMC). The different colours represent the different groups. The times (*x*‐axis) and the effective population size (*y*‐axis) were log_10_ transferred. (b) Maximum likelihood tree for the gene flow and genetic drift amongst four groups of *G. soja*. The *x*‐axis means the strength of genetic drift. The arrows show the gene flows and migration rate derived from source groups.

The above result suggests that environmental factors could be a driving force for the differences in the genetic diversity of the *G. soja* population. We asked whether the different growing habitats affected the demographic history of each *G. soja* group. To evaluate it, we analysed the demographic history by evaluating the expansion time of effective population size of different groups as inferred by the pairwise sequentially Markovian coalescent (PSMC). The PSMC analysis was performed by merging all variants identified from all the accessions within a group due to the selfing feature of *G. soja* (Figure 3a). Demographic history could be traced back to approximately as early as 3 million years ago (Ma), and the population size continues to decrease from 0.6 Ma to 0.2 Ma, which may be caused by low temperature during the Naynayxungla Glaciation. In addition, the demographic history analysis revealed that the four groups of *G. soja* diverged ~1 × 10^5^ years ago (Figure 3a). After then, the effective population sizes for all four groups continued to expand with different degrees. For example, the SR group expanded earlier than other groups, and it expanded more dramatically than the other three groups; and by contrast, the effective population size of the NER.II group was relatively stable, without the observation of significant expansion and divergence within the group (Figure 3a). These results were consistent with the above‐mentioned result where SR retained a higher level of genetic diversity whilst NER.II has the lowest level of genetic diversity.

The variation in genetic diversity and demographic history for the four groups urged us to examine whether any genetic drift occurred in the groups. Maximum likelihood tree as performed by TreeMix analysis showed the earliest divergence happened in between HR‐SR and NER (Figure 3b). Then, HR and SR, NER.II and NER.I diverged, respectively. This observation is consistent with the phylogenetic results where *G. soja* accessions were clustered mainly according to their geographical regions (Figure 1b). Introgression was observed from HR to NER.I, but not NER.II may explain the higher diversity in NER.I group than that in NER.II group. Furthermore, the NER.II group showed the highest level of genetic drift over other groups (Figure 3b), which might be the cause for the lowest genetic diversity in NER.II.

### 3.5 Genome‐environment association revealed genetic loci associated with local adaptation

**FIGURE 4:**
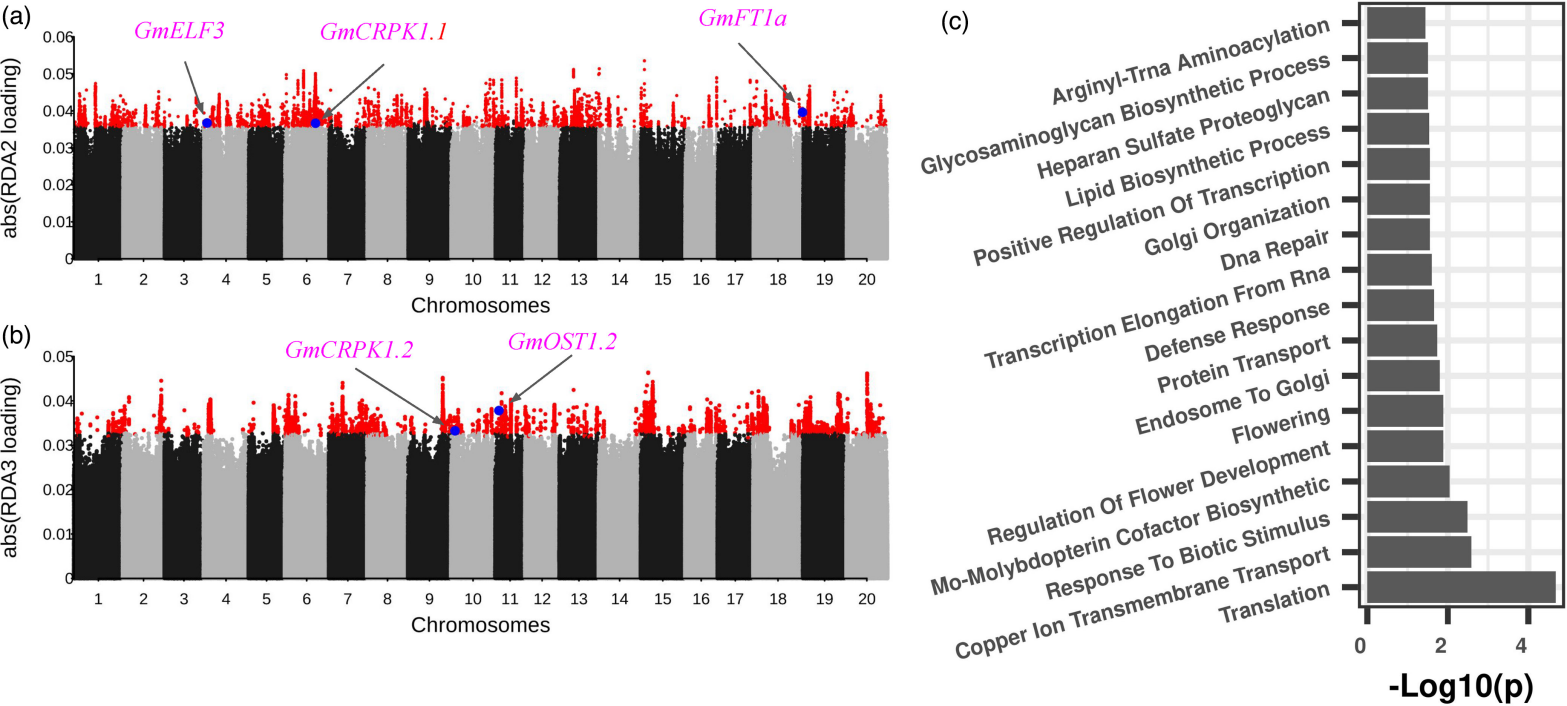
Genome‐wide environment association using redundant analysis. Manhattan plot of the absolute SNPs loadings from (a) RDA2 and (b) RDA3. The *y*‐axis indicates the absolute RDA score for each SNPs. The red points mean the outlier SNPs. The blue points indicated the known genes colocalized SNP. (c) Gene ontology enrichment analysis of genes underlying the outliers from RDA. Overrepresented gene ontology terms were identified using a *p‐*value <0.05.

The above results revealed that the environmental factors played important roles in shaping the genetic diversity and in the divergence amongst four groups of *G. soja*, and the diversity patterns might be tightly associated with local adaptation. To understand the genetic basis of local adaptation, genome‐environment association (GEA) was performed using two methods: RDA and latent factor mixed models (LFMM). There were 43,973 outlier SNPs detected by the RDA‐based genome scan method for five RDAs (Figure 4a,b, Figure S4). Some of those loci were identified for more than one RDA, whilst the other loci were only identified for specific RDA (Table S6). In total, 9863 adaptive genes were extracted from those outliers. GO term enrichment showed those genes were significantly enriched in translation (GO:0006412, *p* = 2.1 × 10^−5^), and adaptation‐related terms, for example, flower development (GO:0009909, *p* = 1.3 × 10^−2^), photoperiodism (GO:0048573, *p* = 1.3 × 10^−2^), and biotic and abiotic stress response terms (Figure 4c). Translation (GO:0006412) is an important process in plant growth and metabolism, which may be regulated during the adaptation to new environments to balance the energy between stress response and plant growth (López‐Maury et al., 2008). Flower development (GO:0009909), photoperiodism (GO:0048573), and biotic and abiotic stress response are important for the photoperiod‐sensitive and cold‐sensitive plants, such as *G. soja*. Adapting to the gradient latitude and day‐length change requires functional changes in the adaptation‐related genes.

**FIGURE 5:**
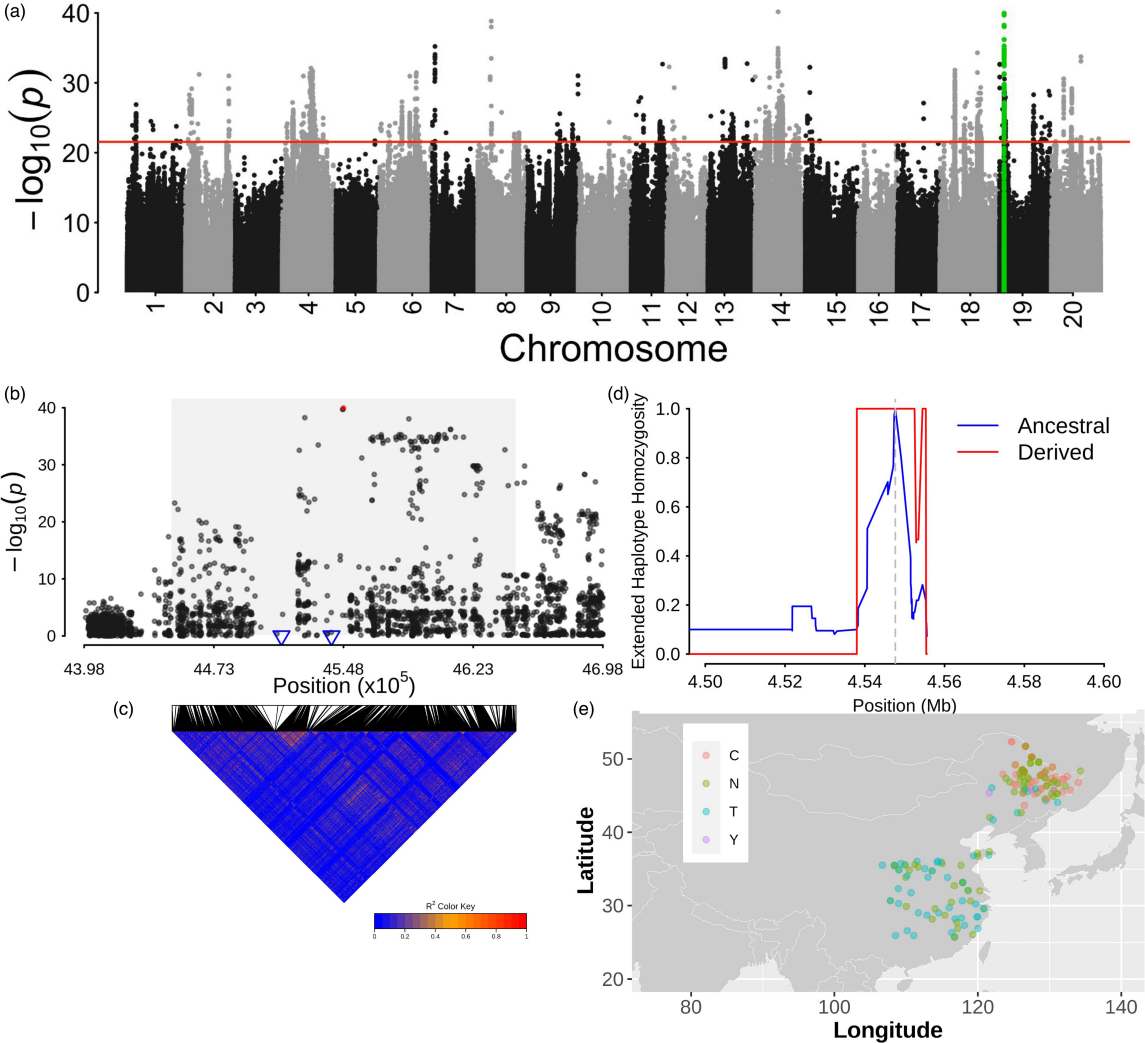
A pleiotropic locus on chromosome 19. (a) Manhattan plot for the genome‐wide environment association for Bio1 using latent factor mixed model. The highlight SNPs with green means the pleiotropic locus. (b) The regional association for the association on chromosome 19. The shadow region indicated the 100 kb region around the leading SNP (rs19_04547654) coded with red. Two blue triangles are two candidates ‘*Glyma.19G034500*’ and ‘*Glyma.19G034600*’. (c) LD heatmap corresponding to the shadow region in (b). The colour from blue to red indicated the LD level from 0 to 1. (d) Extended haplotype homozygosity (EHH) decay around the leading SNP. (e) The alleles distribution of the leading SNP rs19_04547654. Colours mean different alleles. N and Y mean missing and heterozygous alleles at leading SNP.

Using latent factor mixed models (LFMM), a total of 24,368 SNPs colocalized with 4860 genes were identified for three geographic (latitude, longitude and altitude) and five environmental factors (Bio1: annual mean temperature, Bio3: isothermality, Bio12: annual precipitation, Bio13: precipitation of wettest month, Bio15: precipitation seasonality) (Figure 5a, Figure S5). To identify the overrepresented pathways involved in *G. soja* local adaptation from LFMM, GO enrichment analysis was performed for the 4860 genes. We observed the most highly enriched terms were exocytosis (GO:0006887, *p* = 4.1 × 10^−3^), and nicotianamine biosynthetic process (GO:0030418, *p* = 5.5 × 10^−3^) (Figure S6). Exocytosis has been shown to play an important role in response to environmental cues (Zhang, Dong, et al., 2019; Zhang, Xing, & Lin, 2019), and the nicotianamine biosynthetic process was also shown with roles in regulating plant tolerance to abiotic stress (Kim et al., 2005; Nozoye, 2018). Interestingly, comparing the results from RDA, 6324 SNPs and 1726 genes overlapped in both approaches. These few associations overlapping between RDA and LFMM indicate that these two approaches rely on different algorithms, and suggest the necessary use of these two approaches to identify the associations for environmental adaptation.

### 3.6 Flowering time plays an important role in the local adaptation of *G. soja*

*Glycine soja* is a short‐day plant, and the adaptation to different photoperiods requires the changes in flowering‐time genes, which was also supported by the GO enrichment for flowering‐related pathways (Figure 4c). To check whether the previously identified flowering time‐associated genes in cultivated soybean were also involved in *G. soja*'s local adaptation, we searched and found multiple genes with demonstrated functions in the regulation of flowering time and photoperiod response in the RDA and LFMM results. Two outlier SNPs (rs4_4080362, RDA score = −0.0367 in RDA2, and rs4_4075126, RDA score = −0.0207 in RDA5) on chromosome 4 were colocalized with one flowering‐time gene *GmELF3* (*Glyma.04G050200*) (Table 1, Figure 4a, Figure S4). *GmELF3* encodes a hydroxyproline‐rich glycoprotein family protein and it underlies *J* locus, which could extend the vegetative phase under short‐day conditions in southern China (Lu et al., 2017). Besides, the *J* locus also regulates the expression of *FT* genes, such as *GmFT5a* (*Glyma.16G044100*) (Lu et al., 2017). *GmFT5a* plays an important role in regulating the flowering time to adapt to the high‐latitude region in soybean, colocalizing with rs16_4130867 from RDA 5 (RDA score = −0.0209) (Figure S4) (Cai et al., 2020). Another *FT* gene, *GmFT1a*, (*Glyma.18G298900*) colocalized with the outlier SNP rs18_57649585 on chromosome 18 (RDA score = −0.0396 in RDA2) (Figure 4a). *GmFT1a* could delay flowering and maintain vegetative growth in soybean under long‐day conditions (Liu et al., 2018). Similarly, *E2* (*Glyma.10G221500*), a major flowering time regulator in soybean, was colocalized with one SNP (rs10_45295508; *p* = 6.41 × 10^−20^), which was identified using LFMM for longitude (Figure S5). *Phytochrome B* (*PhyB*, *Glyma.15G140000*) was colocalized with the SNP rs15_11428805 (*p* = 1.33 × 10^−5^), which was identified using LFMM for altitude (Figure S5). *PhyB* is a photoperiod‐sensitive gene and plays an important role in the regulation of floral induction (Childs et al., 1997; Mockler et al., 1999). Hence, these results indicated the flowering‐time‐related genes identified in soybean contribute to the latitudinal adaptation of *G. soja*.

**TABLE 1:** Candidates under the outliers from genome‐environment associations

SNPs	RDA score/LFMM *p* value	Methods	RDAs/LFMM Variables	Gene ID	Gene name	Related traits
rs4_4080362	−0.0367	RDA	RDA2	*Glyma.04G050200*	*GmELF3*	Flowering time
rs4_4075126	−0.0207	RDA	RDA5	*Glyma.04G050200*	*GmELF3*	Flowering time
rs16_4130867	−0.0209	RDA	RDA5	*Glyma.16G044100*	*GmFT5a*	Flowering time
rs18_57649585	−0.0396	RDA	RDA2	*Glyma.18G298900*	*GmFT1a*	Flowering time
rs10_45295508	6.41 × 10^−20^	LFMM	Longitude	*Glyma.10G221500*	*E2*	Flowering time
rs15_11428805	1.33 × 10^−5^	LFMM	Altitude	*Glyma.15G140000*	*PhyB*	Flowering time
rs6_36027725	−0.0366	RDA	RDA2	*Glyma.06G230800*	*GmCRPK1.1*	Cold
rs10_4741222	0.0333	RDA	RDA3	*Glyma.10G052500*	*GmCRPK1.2*	Cold
rs10_4741329	−0.0222	RDA	RDA4	*Glyma.10G052500*	*GmCRPK1.2*	Cold
rs11_4449508	0.0378	RDA	RDA3	*Glyma.11G058800*	*GmOST1.2*	Cold
rs10_24593415	1.62 × 10^−8^	LFMM	Bio3	*Glyma.10G108000*	*GmOST1.1*	Cold
rs6_13677255	2.03 × 10^−5^	LFMM	Altitude	*Glyma.06G165000*	*GmICE1*	Cold
rs14_7454002	1.04 × 10^−7^	LFMM	Bio3	*Glyma.14G084700*	*GmDREB1B;1*	Cold

Abbreviations: LFMM, latent factor mixed model; RDA, redundancy analysis.

### 3.7 Cold‐tolerance‐related genes were involved in the local adaptation of *G. soja*

Due to the temperature difference between the northern and southern regions, there may be temperature‐responsive genes affecting adaptation. We observed that multiple cold‐tolerance‐related genes were colocalized with the outlier SNPs identified from both RDA and LFMM. One outlier SNP (rs10_24593415, *p* = 1.62 × 10^−8^) on chromosome 10 for isothermality (Bio3) from LFMM was physically close to *GmOST1.1* (*Glyma.10G108000*) (Table 1, Figure S5), and the other outlier on chromosome 11 from RDA3 (rs11_4449508, RDA score = 0.0378) was colocalized with *GmOST1.2* (*Glyma.11G058800*) (Figure 4b). Both genes were orthologs of *AtOST1*, belonging to the protein kinase superfamily protein, which can increase cold tolerance by enhancing the stability of ICE1, a central role in regulating freezing tolerance in *Arabidopsis* (Chinnusamy et al., 2003; Ding et al., 2015). Coincidentally, *Glyma.06G165000*, an ortholog of *AtICE1*, was colocalized with the outlier (rs6_13677255, *p* = 2.03 × 10^−5^) for altitude on chromosome 6 (Figure S5). In addition, two genes that are orthologs of *AtCRPK1*, which codes a phosphorylates 14‐3‐3 protein and fine‐tunes C‐repeat‐binding factor (CBF) signalling during cold tolerance (Liu et al., 2017), were also identified in the analysis, including *Glyma.06G230800* (rs6_36027725, RDA2 score = −0.0366) (Figure 4a) and *Glyma.10G052500* (rs10_4741222, RDA3 = 0.0333 and rs10_4741329, RDA4 score = −0.0222) (Figure 4a, Figure S4). *GmDREB1B;1* was colocalized with the SNP (rs14_7454002, *p* = 1.04 × 10^−7^) (Figure S5), which was identified for isothermality (Bio3) using LFMM. In soybean, *GmDREB1B;1* (*Glyma.14G084700*) was strongly induced under cold, drought or other abiotic stresses (Kidokoro et al., 2015) and its *Arabidopsis* ortholog (*AT1G46768*) is induced during cold tolerance and confers cold tolerance in *Arabidopsis* (Dong & Liu, 2010; Yamasaki & Randall, 2016).

### 3.8 A pleiotropic locus on chromosome 19

We identified a locus which was identified in both approaches for multiple variables on the short arm of chromosome 19 around 4.5 Mb (Figure 5a, Figures S4, S5), including RDA1, Bio1, Bio3, Bio13, Bio15, longitude and latitude. This result suggests the pleiotropic effect of this locus on different environmental factors. The locus contains the leading SNP (rs19_04547654) association for Bio1, and it was colocalized within *Glyma.19G034600*, which encodes an *AGAMOUS‐like 8* (*AGL8*) gene (Figure 5a). Ten annotated genes were detected in the 100 kb interval around the leading SNP based on the reference genome. Amongst the ten genes, two genes (*Glyma.19G034500* and *Glyma.19G034600*) were associated with flower development (Figure 5b). Both genes were tandem duplicated and localized on the same LD block harbouring the leading SNP (Figure 5c). The extended LD analysis demonstrated the positive selection on the locus by the longer LD block for the derived allele (Figure 5d), which may be a result of local adaptation. Considering the locus underlying the adaptation to latitude and temperature, we asked whether the alleles of the locus differentiated geographically. To test it, we mapped the leading SNP (rs19_04547654) on the geographic map; the analysis showed a clear geography‐oriented distribution. Specifically, allele ‘C’ was highly concentrated in NER, whilst allele ‘T’ was identified in all the selected regions (NER, HR and SR) (Figure 5e). The allele distribution further supported that positive selection occurred on the locus, suggesting the important role of the allele ‘C’ in the high‐latitude region adaptation.

Both flower development‐related genes, *Glyma.19G034500* (*GmSEP1*) and *Glyma.19G034600* (*GmFUL*), encode MADS‐box transcription factor family protein with K‐box region. *GmSEP1*, an ortholog of the *Arabidopsis AtAGL4* (*AT3G02310*), has been validated to play an important role in reproductive development, specifically in petal, and seed coat development in soybean (Huang et al., 2009). *GmFUL* codes an Agamous‐like MADS‐box protein AGL8. Its ortholog (*AtFUL: AT5G60910*) in *Arabidopsis* regulates flowering time and reproductive organ size (Karami et al., 2020; Marzo et al., 2020). Because both genes are involved in flower development, they may change their expression pattern to adapt to the different day lengths. To detect their response to photoperiod, we used the expression data from a previous study that sequenced the *G. soja* under different day lengths (GEO accession: GSE51007) (Wu et al., 2014). We found that under short day (SD, 10‐h light: 14‐h dark), *Glyma.19G034600* is highly expressed at 6:30, declined sharply over the next eight hours until to 14:30, then increased over the next 8 h (22:30 in the evening), whereas no expression was detected under long‐day condition (16‐h light: 8‐h dark) (Figure S7). Similar to *Glyma.19G034600*, *Glyma.19G034500* showed expression under SD, and extremely low expression was observed under long‐day conditions. The results suggested these two genes are high‐confidence candidates for *G. soja* adaptation.

## 4 DISCUSSION

### 4.1 Local adaptation shaped the diversity pattern in *G. soja*

Natural selection is the major driver of evolution and divergence within species. It leads to the divergence of the same species under different environments, known as local adaptation (Savolainen et al., 2013). *Glycine soja* is widely distributed from low to high latitude in East Asia, and it possesses the ability to adapt to a wide range of diverse environments. These diverse environments may result in the different selection forces for *G. soja* in different locations. In this study, the phylogenetic analysis together with model‐based clustering and PCA has demonstrated that the divergence of *G. soja* was geography oriented, which is in agreement with the ecological zones in China, including Southern, central and Northeast regions (He et al., 2016; Wen et al., 2009). *Glycine soja* in Northeast China is exposed to long‐day length and low temperature. By contrast, *G. soja* in Southern China is exposed to short‐day length and warm temperature. The opposite environments have selected the genomic diversity in different directions, which shaped the geographic structure of *G. soja* and led to local adaptation. Intriguingly, the *G. soja* in the Northeast region was further separated approximately along the latitude of 48° N. Given the fact that 48°N was roughly on the separation line of the cold‐temperate zone and mid‐temperate zone of China (Zheng et al., 2010), the NER.I and NER.II groups might have different traits to adapt to the local environments.

### 4.2 Demographic history is intimately related to ecological environments

Quaternary (2.4 million years ago to the present) climatic changes affected the demography and distribution of plants (Hewitt, 2000, 2004). In our study, the different groups of *G. soja* diverged and expanded during the last glacial period (LGP) (1 × 10^5^–1 × 10^4^ years ago). However, we did not detect significant bottlenecks in *G. soja* during LGP, and this finding was supported by previous studies (He et al., 2016; Kim et al., 2021; Zhou et al., 2015). There are two possibilities for how the *G. soja* population expanded and diverged during the LGP. One possibility is that *G. soja* survived in multiple cryptic refugia in China during the last glacial maximum (2.2 × 10^4^ years ago) and began to expand and diverged due to the heterogeneity of the environment (Leamy et al., 2016). It is supported by a previous study that Northeast China and the Yangzi River basin (middle and downstream of the Yangtze River) were refugia for *G. soja* (Leamy et al., 2016). The other possibility is that the main refugia of *G. soja* is mainly in southern China during the LGM. Then, *G. soja* gradually expanded from the southern to the northern region. It is supported by the previous study that *G. soja* was inferred to be limited to southern and central China during the LGM and experienced large‐scale post‐LGM range expansion into northern East Asia (He et al., 2016). During the process, the climate differences in the southern and northern regions led to the differentiation of *G. soja*. In the southern region, the warmer temperature and richer precipitation were suitable for the growth of *G. soja*; therefore, the population of *G. soja* expanded more rapidly. We are more inclined to support the latter inference. First, as a short‐day plant, *G. soja* was more likely to originate from lower latitudes in China (Gai et al., 2000; Summerfield et al., 1985; Summerfield & Roberts, 2018). Second, the southern group showed a higher level of nucleotide diversity than the northeast group (Wen et al., 2009).

### 4.3 Multiple pathways involved in the local adaptation of *G. soja*

*Glycine soja* has a broad distribution, spanning 24°N and 53°N latitude in East Asia. Environments vary extensively across their range, with altitude ranging from 0 to 2670 metres, yearly precipitation ranging from 300 mm to 2300 mm, and mean annual temperature ranging from −3.1°C to 18.2°C (Anderson et al., 2016). The adaptation of *G. soja* to these diverse environments requires selection on various fitness‐related traits, such as flowering time, seed amount, growth patterns, biotic and abiotic stress tolerance (Kofsky et al., 2018; Lu et al., 2020). Most adaptive traits have a polygenic genetic basis, that is, the genetic architectures are determined through shifting the allele frequencies at many loci (Barghi et al., 2020). Consistently, our results suggest that the diverse pathways were involved in natural selection. Those pathways include genes in expression regulation, flower development and different stress responses. To effectively respond to the different environments, the plant would regulate the expression of adaptive traits‐related genes to balance between the fitness and growth (López‐Maury et al., 2008). For example, we observed the translation‐related terms were enriched in adaptation, which is an important term related to plant growth and development. Furthermore, we found several candidate loci associated with environmentally adaptive traits, including the well‐characterized flowering‐time genes, such as *GmELF3*, *GmFT1a*, *GmFT5a* and several cold‐tolerance‐related loci in soybean. These associated pathways and genes indicated multiple pathways were involved in the local adaptation of *G. soja*, which implies the polygenic adaptation. Some of these genes have been proved to play an important role in the adaptation of *G. max*. For example, the loss‐of‐function alleles of *GmELF3* were highly enriched in the low‐latitude adaptation of soybean (Lu et al., 2017). Whether these genes have a critical role in the adaptation of *G. soja* remains for further functional verifications.

### 4.4 The power of RDA and LFMM in genome‐environment association

The two approaches were widely used for identifying the genome‐environment associations. As the adaptive loci largely vary under the different selection pressures or mechanisms, different methods may help to identify the selection loci caused by various forces or selection mechanisms (Forester et al., 2018). LFMM is a univariate genotype‐environment association method, which might have a low detection rate for loci under weak selection (Forester et al., 2018). By contrast, RDA is a multivariate statistical method based on constrained ordination; it can detect adaptations that result in weak, multilocus selections (Forester et al., 2018; Rellstab et al., 2015). Previous studies have shown that RDA performed better than LFMM in detecting adaptation loci (Capblancq et al., 2018; Forester et al., 2018). In our study, different loci were identified using two approaches. Using RDA, genes related to the well‐characterized adaptive traits, such as flowering time and photoperiodism, were identified under natural selection (Romero Navarro et al., 2017; Sedivy et al., 2017). However, LFMM has limited power to identify those loci compared with RDA, because those variations were highly correlated with the population structure that was controlled in LFMM. The different GO enrichment results from RDA and LFMM indicated that the RDA had higher power in identifying the loci under selection (Forester et al., 2018).

### 4.5 Conclusion

In summary, we collected 185 *G. soja* accessions from three major agro‐ecological zones in China and analysed genomic diversity to investigate the genetic basis of local adaptation using the whole‐genome sequencing data. We revealed that *G. soja* exhibited clear geographic population structure and multiple environmental factors contribute to the genetic differentiation. The demographic history analysis showed *G. soja* from the three ecological zones diverged about 1 × 10^5^ years ago, and then its effective population sizes have undergone different degrees of expansions. Genome‐wide environment associations identified multiple genes involved in the local adaptation of *G. soja*, especially the flowering time and temperature‐related genes. Lastly, the present study elaborates on the genetic basis of the local adaptation of *G. soja* and provides new insights into the *G. soja* divergence, which is helpful for breeding climate‐resilient soybean varieties in wider regions beyond the current major soybean cultivation areas.

## CONFLICT OF INTEREST

The author declares that there is no conflict of interest.

## Supporting information


Figures S1‐S7

Tables S1‐S6

## Data Availability

The data that support the findings of this study are openly available in NCBI under accession number PRJNA597660.
