# Supplementary material for: Whole‐genome resequencing reveals signature of local adaptation and divergence in wild soybean
Source: Evol Appl. 2022 Sep 20;15(11):1820–33. doi: 10.1111/eva.13480 (PMC9679240; doi:10.1111/eva.13480)
Supplement: Supplementary file 1 — Figures S1‐S7 [file EVA-15-1820-s001.docx]

**
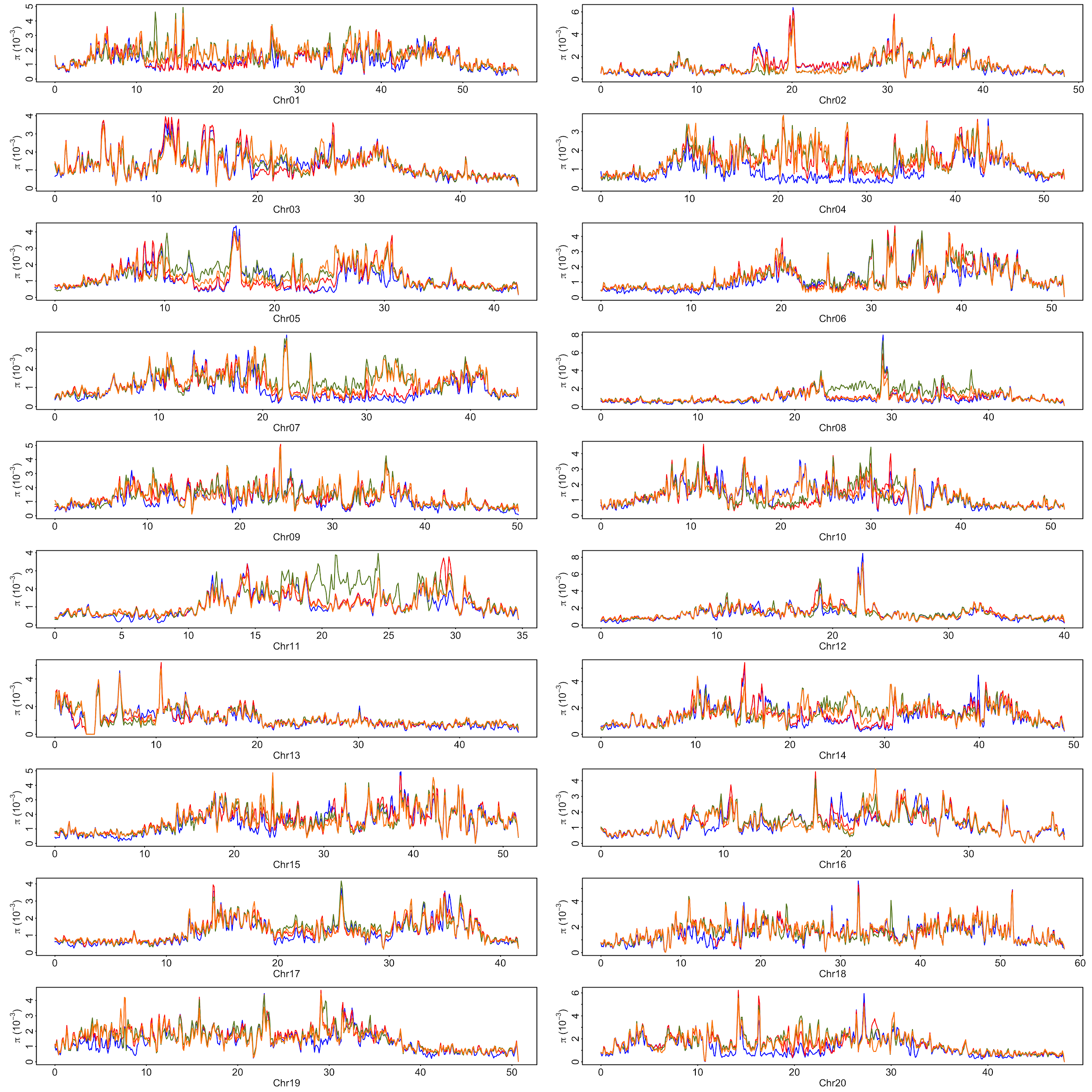
**

**Suppl. Fig. 1.** Nucleotide diversity (π) across 20 chromosomes in four groups. Different colors represent different groups. Blue, red, green and orange represent NER.II, NER.I, HR, and SR respectively.


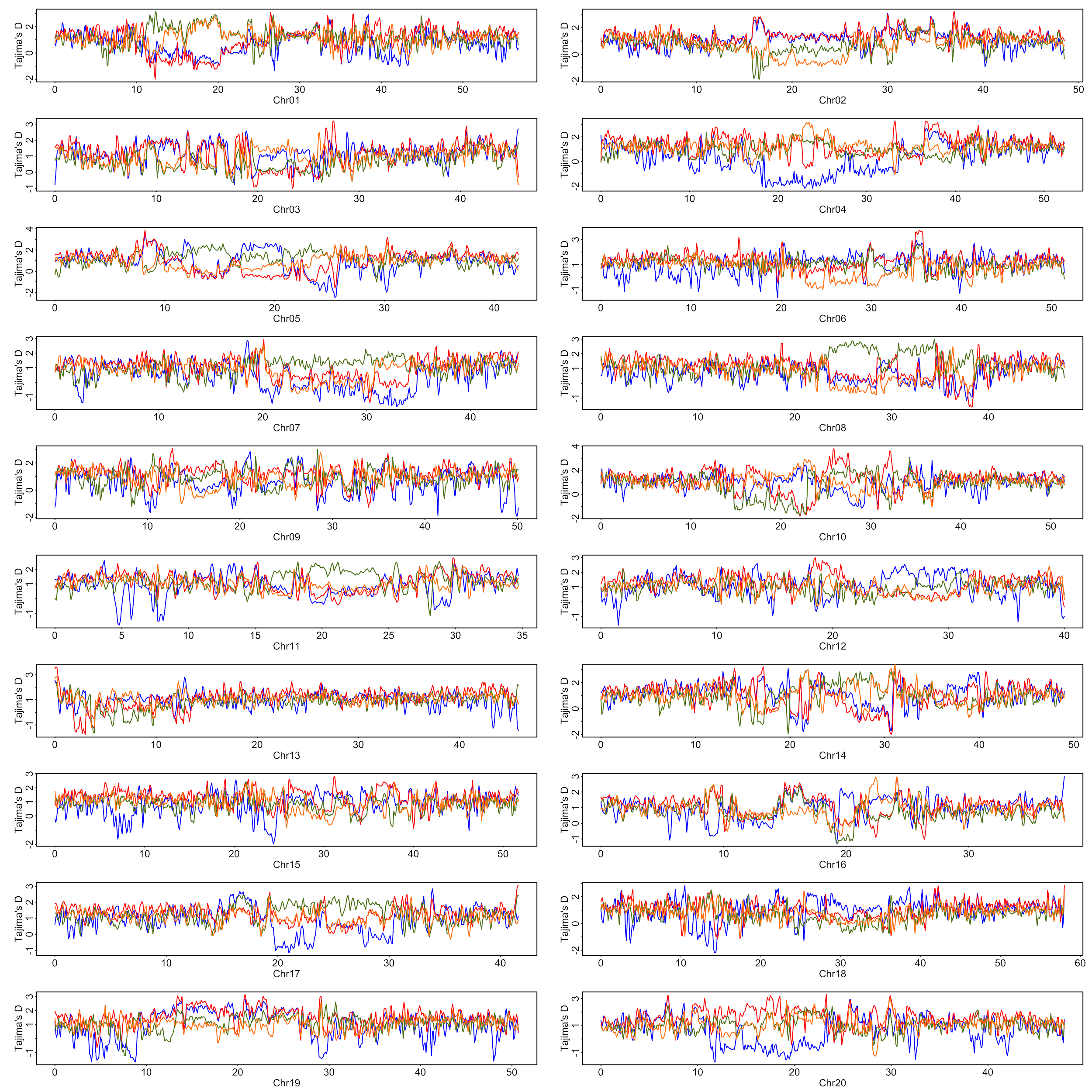


**Suppl. Fig. 2.** Genome-wide Tajima's *D* across 20 chromosomes in four groups. Different colors represent different groups. Blue, red, green and orange represent NER.II, NER.I, HR, and SR respectively.


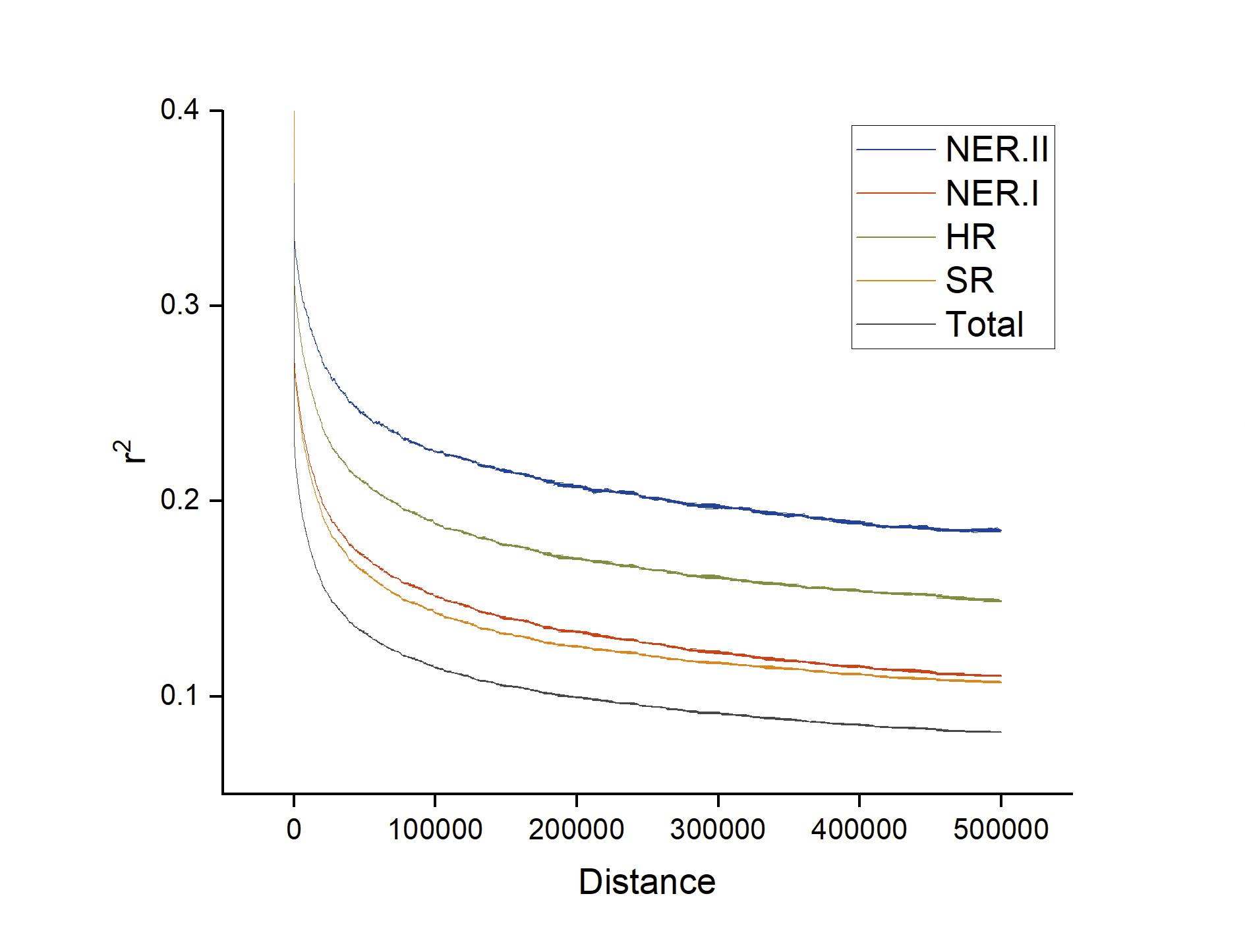


**Suppl. Fig. 3.** LD decay determined by *r*^2^ in four groups and all wild soybean accessions. The different colors mean the LD decay for each subgroup.


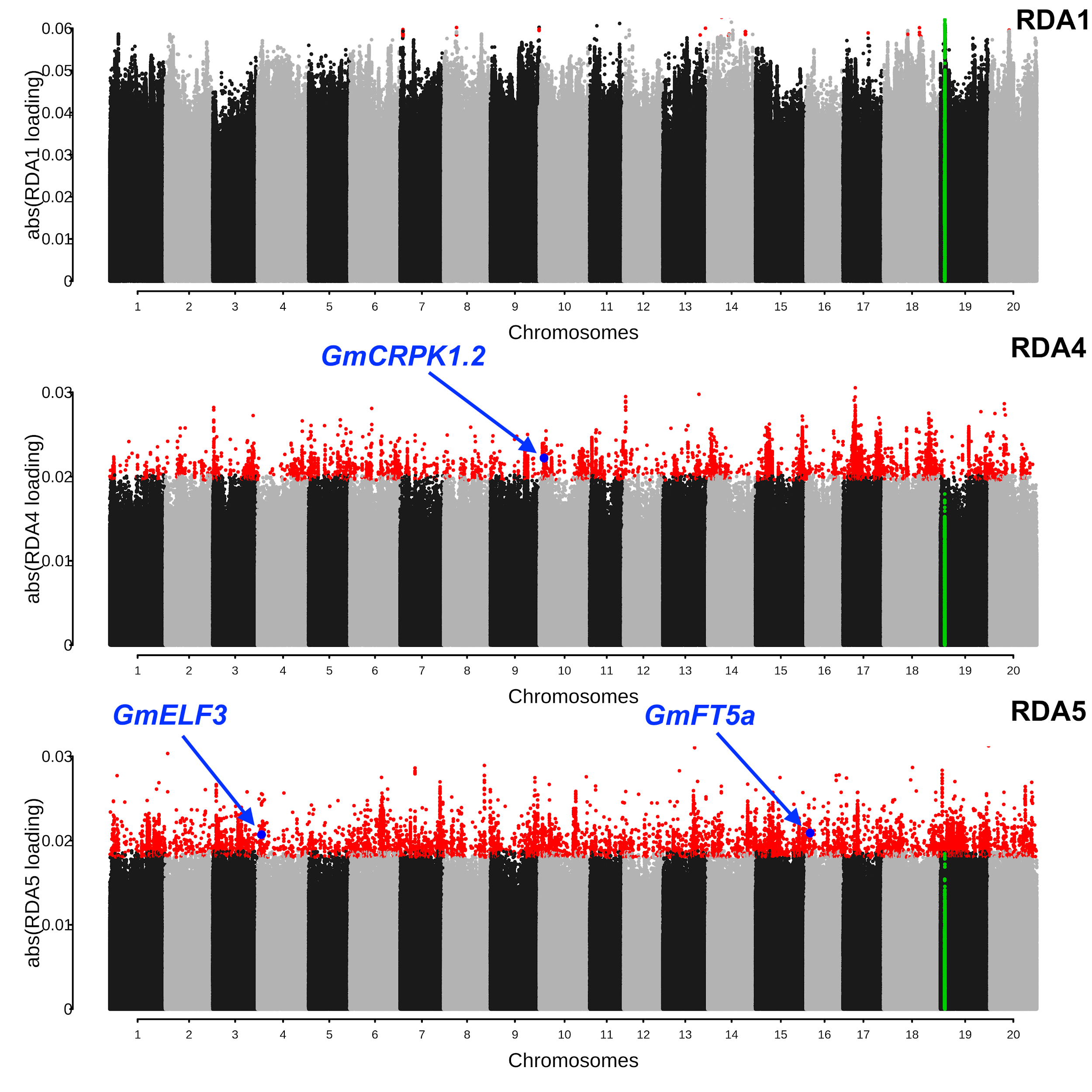


**Suppl. Fig. 4.** Genome-environment association identified candidate SNPs of local adaptation. Manhattan of redundancy analysis (RDA) for (A) RDA 1, (B) RDA 4 and (C) RDA5. The y-axis indicates the absolute RDA score for each SNPs. The red dots signify the outlier SNPs. The blue dot means the SNPs with known functional genes; the green SNPs indicate the genomic region for the pleiotropic locus.


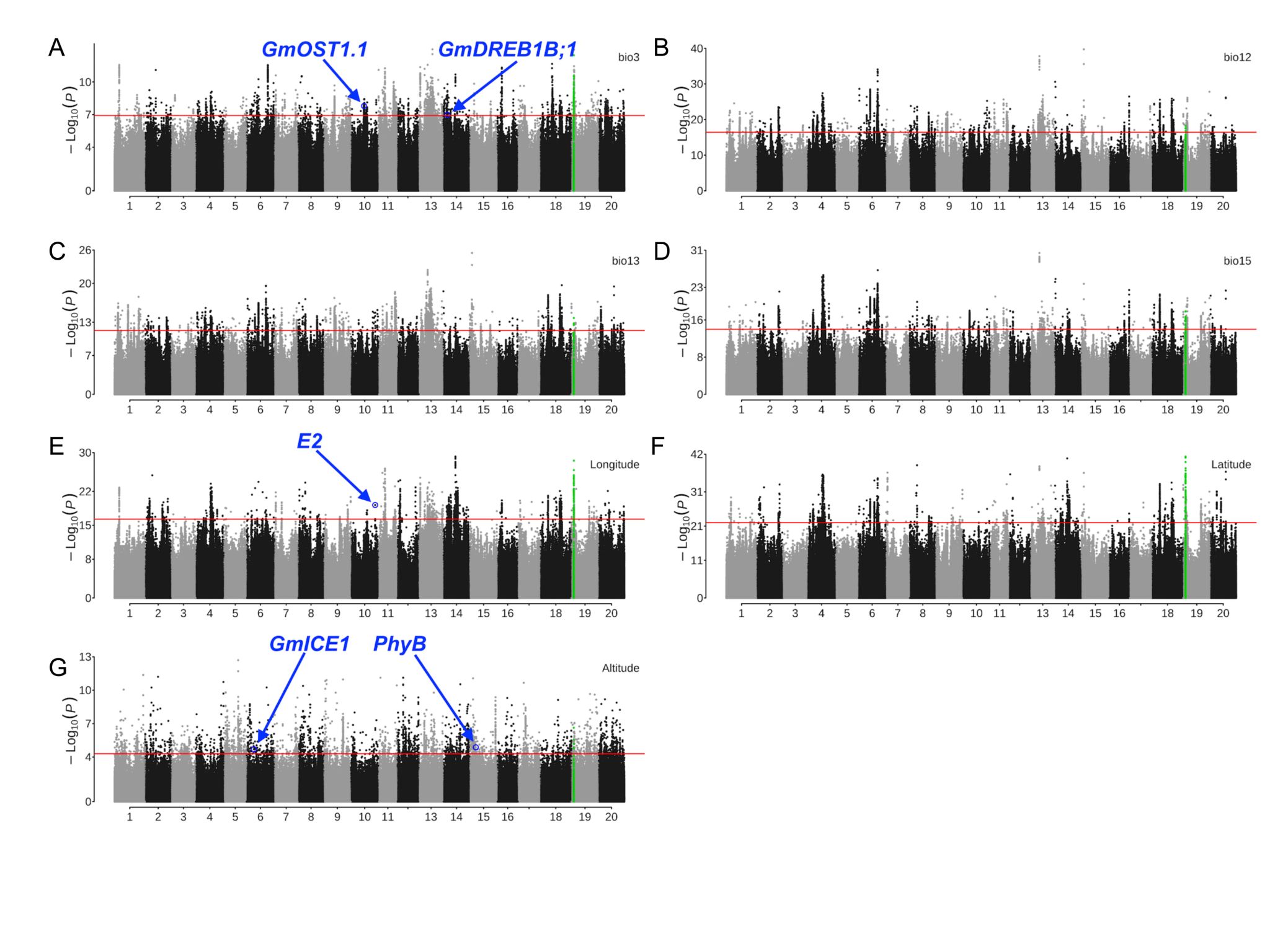
**Suppl. Fig. 5.** Manhattan plot for the genome-wide environment association using latent factor mixed model. (A) Bio3, (B) Bio12, (C) Bio13, (D) Bio15, (E) Longitude, (F) Latitude, (G) Altitude. The blue dot means the SNPs with known functional genes; the green SNPs indicate the genomic region for the pleiotropic locus.


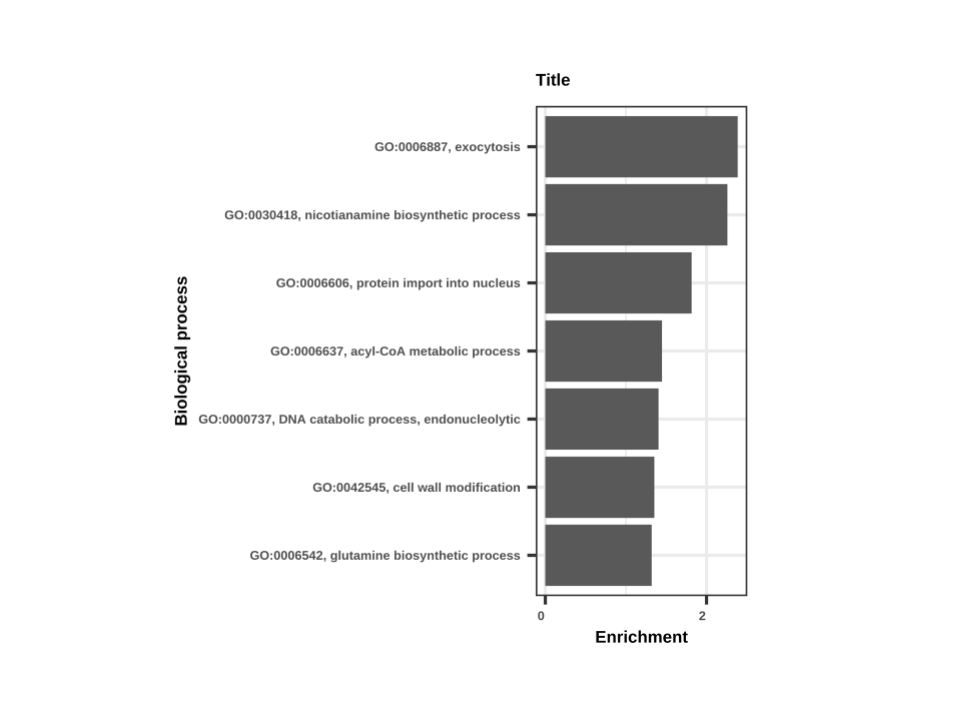


**Suppl. Fig. 6.** Gene ontology enrichment analysis of genes underlying the outliers using latent factor mixed model. x-axis (Enrichment) means -log_10_ (*p*).


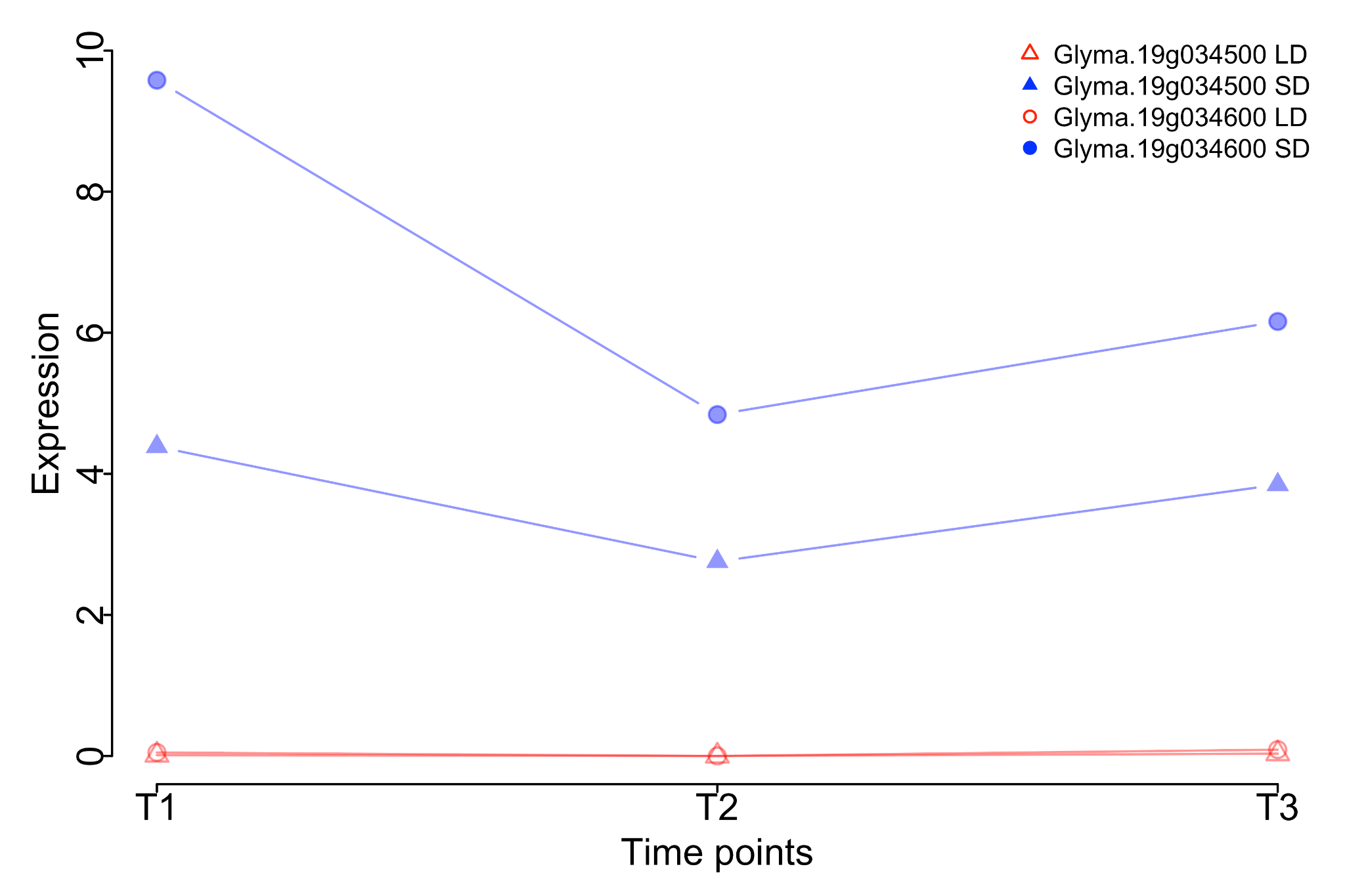


**Suppl. Fig. 7.** The expression of the two candidate genes. Short days (SD) are 10 hours light (6:45–16:45) and long days (LD) are 16 hours light (6:45–22:45). T1, T2, and T3 represent the time points at 6:30, 14:30, and 22:30.
